# Mice modulate ultrasonic calling bouts according to sociosexual context

**DOI:** 10.1098/rsos.180378

**Published:** 2018-06-20

**Authors:** Yui K. Matsumoto, Kazuo Okanoya

**Affiliations:** 1 Department of Life Sciences, Graduate School of Arts and Sciences, The University of Tokyo, 3-8-1 Komaba, Meguro-ku, Tokyo 153-8902, Japan; 2 Department of Functional Brain Research, National Institute of Neuroscience, National Center of Neurology and Psychiatry, 4-1-1 Ogawa-Higashi, Kodaira, Tokyo 187-8502, Japan

**Keywords:** ultrasonic vocalizations, communication, social behaviour, mouse

## Abstract

Mice produce various sounds within the ultrasonic range in social contexts. Although these sounds are often used as an index of sociability in biomedical research, their biological significance remains poorly understood. We previously showed that mice repeatedly produced calls in a sequence (i.e. calling bout), which can vary in their structure, such as Simple, Complex or Harmonics. In this study, we investigated the use of the three types of calling bouts in different sociosexual interactions, including both same- and opposite-sex contexts. In same-sex contexts, males typically produced a Simple calling bout, whereas females mostly produced a Complex one. By contrast, in the opposite-sex context, they produced all the three types of calling bouts, but the use of each calling type varied according to the progress and mode of sociosexual interaction (e.g. Harmonic calling bout was specifically produced during reproductive behaviour). These results indicate that mice change the structure of calling bout according to sociosexual contexts, suggesting the presence of multiple functional signals in their ultrasonic communication.

## 1. Introduction

**Figure 1.**
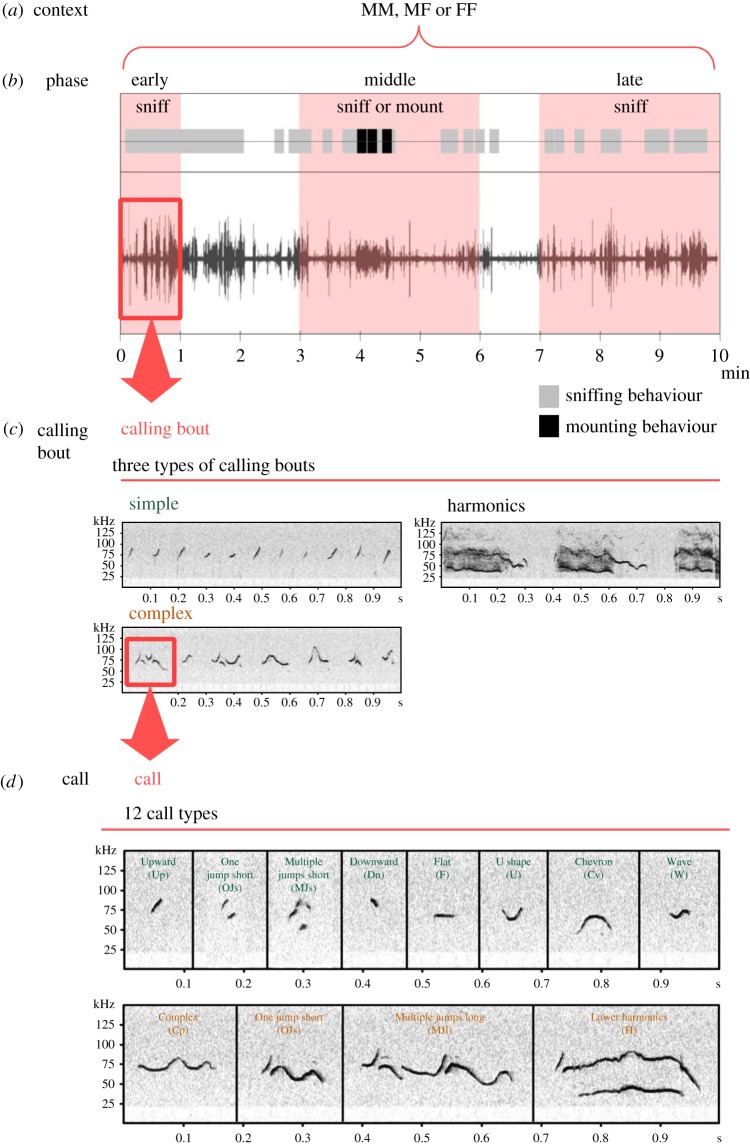
Schematic diagram and representative spectrogram showing the contexts, phases, calling bouts and calls in the present study. (*a*) Context: in this study, we recorded vocalizations in three social contexts; male–male (MM), male–female (MF) and female–female (FF). (*b*) Phase: we separated each context into three phases according to interaction time: early, middle and late phases. We grouped the middle phase in the MF context into two phases: middle phase featuring sniffing behaviour and middle phase featuring mounting behaviour. We did this because several mice displayed not only sniffing but also mounting behaviour in the middle phase (see Material and methods). (*c*) Calling bout: calling bouts are clusters of vocalizations that feature unique proportions of call types, separated by phase in this study. We compared calling bouts produced in the different phases of the social contexts. In our previous study, we found that mice produced three types of calling bouts in an MF context; Simple, Complex and Harmonics. (*d*) Call: calls are individual sounds, separated by silence. Call classification was adapted from a previously published criterion [18]. The upper panel shows short calls (mean duration less than 60 ms) and the lower panel shows long calls (mean duration ≧ 60 ms).

Mice produce ultrasonic vocalizations in a variety of social contexts, such as between a mother and pups, mother and father, and between adult mice. Ultrasonic vocalizations have been a particular focus of attention because Holy & Guo [1] showed that vocalizations in male–female (MF) mouse contexts consist of multiple vocal elements, similar to a song (figure 1). These mouse vocalizations in an MF context are considered to be ‘courtship’ songs because adult males produce most of the vocalizations in response to females or female urine [2–5]. Additionally, these vocalizations have been found to attract

female mice [6–9]. The proportions of several call types depend on the mouse strain [10–13], and females prefer vocalizations produced by a different strain to those produced by same strain of mice [14]. In addition, the rearing environment of the male mouse and the state of the female mouse influence the complexity of the vocalizations in MF contexts, and females prefer complex to simple vocalizations [3,10,11]. Thus, the structure of vocalizations appears to be an important factor in courtship, such that females use it for mate choice. Alternatively, another hypothesis states that mouse vocalizations function not only as ‘courtship’ songs produced by a male for a female, but also as ‘territorial’ songs delivered by a resident to an intruder. Previous studies have indicated that both male and female mice produce vocalizations in same-sex interactions, and that females produce a large number of ultrasonic vocalizations directed towards other females, with structures similar to vocalizations produced in an MF context [2,15–17]. To reveal the functional role of mouse ultrasonic vocalizations, vocalization differences depending on contexts should be clarified.

The relationship between structure and context in mouse vocalizations has been difficult to discern due to difficulty classifying the vocalizations. Mouse ultrasonic vocalizations include various calls, and it can be difficult to detect changes in the vocalizations produced during a social context using only call classification. Although many previous studies have analysed mouse vocalizations, they often regarded the context without considering phases of social interaction. We therefore conducted an acoustic analysis depending on the phase of an opposite-sex context, where we defined a calling bout as a cluster of vocalizations featuring the proportion of a specific call type (figure 1*b*,*c*). Previously, we found that mice produce three types of calling bouts (Simple, Complex and Harmonics; figure 1*c*) according to the interaction time and male sexual behaviour in MF contexts [18]. As the interaction time increases, mice tend to change their vocal production from Simple to Complex calling bouts. They eventually produce calls with a longer duration and higher degree of frequency modulations (Harmonics) during mounting behaviour. Thus, mouse social contexts appear to have several phases, such as communication of territory, approach and reproduction, and phase-specific calling bouts seem to play important roles in each phase [18]. Several previous studies reporting that mouse vocalizations changed depending on behaviour support this suggestion [3,19,20]. Thus, analysis of calling bouts may be used to clarify differences in ultrasonic vocalizations among sociosexual contexts. A previous study reported similarities and differences between male and female mouse ultrasonic vocalizations using call classification [2,16]. Comparing vocalizations according to the phases of context may provide a new foundation for understanding these similarities and differences.

Based on the findings of previous studies, including our study on the relationship between structure and phase of mouse vocalizations, we hypothesized that mice produce several types of calling bouts that vary depending on the context phase (defined by the contact duration and behaviour), and that several phases and phase-specific calling bouts overlap among sociosexual contexts. To address this hypothesis, we recorded mouse vocalizations in male–male (MM), male–female (MF) and female–female (FF) contexts (figure 1*a*). We compared the vocalizations produced in each social context, then examined the vocalizations produced during three or four phases of social interaction and finally examined vocalizations between individuals.

## 2. Material and methods

### 2.1. Animals

Experimental animals were C57BL/6 J mice aged 12–18 weeks (Japan SLC, Hamamatsu, Japan). Mice (males: *n* = 12, females: *n* = 12) were housed individually throughout the experiment in Plexiglas cages (16 cm × 23 cm × 12 cm) with standard bedding (paper chips). Cages were kept in a controlled environment at 22 ± 2°C, with a 12 L : 12 D cycle (lights off at 13.00). Food and water were provided ad libitum.

Exposure mice (males: *n* = 8, females: *n* = 8; 10–20 weeks old) were housed in groups of four. To control oestrus cycle in females, females were ovariectomized under sodium pentobarbital anaesthesia (64.8 mg ml^−1^, quintuple dilution), received an intraperitoneal injection of 17β-estradiol-3-benzoate (approx. 98%, 2 mol l^−1^; Wako Pure Chemical Industries, Osaka, Japan) and received progesterone (approx. ≥99%, 500 µg/0.1 ml; Sigma-Aldrich Japan, Tokyo, Japan) 9 days, 2 days and 1 h before the recording test, respectively.

### 2.2. Apparatus

The mice were tested in Plexiglas cages (20 × 20 × 20 cm) placed in a sound-attenuating chamber (50 × 35 × 35 cm; Muromachi Kikai, Tokyo, Japan). A silicon rubber cover was placed on the bottom of the test cage to decrease noise generated by animal movement. A plastic board (20 × 28 cm) with a hole (1 × 1 cm) in the lower edge was used to separate the test box for the indirect interaction test. The chamber was illuminated by a small red light. Mouse vocalizations and behaviours during the recording test were recorded and monitored by a microphone (UltraSoundGate CM16/CMPA; Avisoft Bioacoustics, Berlin, Germany) and a camera (Adafruit TTL serial camera; Adafruit Industries, New York, NY, USA) that were positioned 30 cm above the floor of the test cage. The microphone was sensitive to sounds in the range of 10–180 kHz. There were no noises generated by the recording apparatus. The vocalizations were recorded using the Avisoft recorder software with a sampling rate of 300 kHz.

### 2.3. Experimental procedure

Mouse vocalizations in MM, MF and FF contexts were recorded using the same paradigm. Animals were handled and habituated to the test cage for 10 min d^−1^ during the dark cycle (14.00–20.00) for 5 consecutive days. Experimental males and females were housed individually. Recordings in all contexts were conducted on 4 consecutive days for each context, once per day, during the dark cycle (14.00–20.00). The MM and MF contexts involved the same group of male mice. The MM and MF experiments were spaced at least 3 days apart, and the test order was counter-balanced. To ensure that the male mice were sexually experienced prior to the experiment, experimental males were housed with exposure females for 3 days and then returned to individual housing 1 day before the recording test (MM and MF context). Thus, the male mice underwent 5 days of cage habituation, 3 days of sexual exposure and 4 days of MM (or MF). Three days later, they underwent MF (or MM). The female mice in FF contexts underwent the same series of manipulations, but did not undergo a sexual exposure period (FF context). Instead, they continued to be housed individually for the 3 days following cage habituation.

On test days, each experimental mouse was placed in the test cage 10 min before the recording test. The test cage was separated into two equally sized sections via a clear plastic wall. The mice were placed in the test cage such that an exposure mouse and an experimental mouse were on opposite sides of the cage. The exposure mice were male in the MM context and female in the MF and FF contexts. Then, a 5 min recording session was conducted (indirect interaction test). At the end of the indirect interaction test, the wall was removed from the test cage and a 10 min recording session was conducted (direct interaction test). Each pair of mice interacted only once across all tests conducted and housing combinations prior to the tests. The order of the recording tests was randomized each day.

### 2.4. Ultrasound analysis

Spectral analyses of the vocalizations were performed using SASLab Pro v. 1.7.1 (Avisoft Bioacoustics, Berlin, Germany) with Windows 7. Sound spectrograms were generated with a fast Fourier transform length of 512 points and Hamming style window with 50% overlap. The spectrograms had a frequency resolution of 977 Hz and a time resolution of 2 ms. The data from the test in which the mice produced the greatest number of vocalizations were used for analyses because bout analysis requires a large number of vocalizations. In female, a number of vocalizations are affected by the oestrus cycle, which is 4–5 days long [21,22]. Female vocalizations in this study, therefore, could occur at any phase of the cycle. Series of calls produced during social contact (sniffing and mounting) were analysed, and sparse calls and those that overlapped were excluded. Sounds with frequencies below 25 kHz were removed to reduce the effects of background noise outside the relevant frequency band. In addition, sounds produced by activities, such as scratching and locomotion in the relevant frequency band, were removed using the ‘eraser’ function. Acoustic features (mean call duration, root-mean-square amplitude (RMS), peak frequency, fundamental frequency, bandwidth and entropy) were measured using ‘automatic measurements'. The vocalizations were categorized into 12 types (figure 1*d*) based on call duration and overall shape, adapted from previously published criteria [18].

We used the following call classifications:

*Upward (Up)*. Calls with an upsweep frequency change (greater than 5 kHz).
*One jump short (OJs)*. Calls with one frequency jump (lasting less than 60 ms).
*Multiple jumps short (MJs)*. Calls with two or more frequency jumps (lasting less than 60 ms).
*Downward (Dn)*. Calls with a downsweep frequency change (greater than 5 kHz).
*Flat (F)*. Calls with a minimal frequency change (less than or equal to 5 kHz).
*U shape (U)*. Calls with a downsweep (greater than 5 kHz) followed by an upsweep (greater than half of the frequency change of the downsweep).
*Chevron (Cv)*. Calls with an upsweep (greater than 5 kHz) followed by a downsweep (greater than half of the frequency change of the upsweep).
*Wave (W)*. Calls with two phases and a change in frequency (greater than 5 kHz).
*Complex (Cp)*. Calls with three or more phases and changes in frequency (greater than 5 kHz).
*One jump long (OJl)*. Calls with one frequency jump (lasting more than or equal to 60 ms).
*Multiple jumps long (MJl)*. Calls with two or more frequency jumps (lasting more than or equal to 60 ms).
*Lower harmonics (H)*. Calls with one or more harmonic sounds (fundamental frequency approx. 30–40 kHz).

Using our previous study [18] as a reference, we investigated changes in the acoustic features and proportions of each call type in the vocalizations with respect to time (early, middle and late phases) and behaviour (sniffing and mounting). Early, middle and late phases corresponded to behaviours that took place in the first minute, 3–6 min, and 7–10 min of contact with the intruder, respectively (figure 1*b*). In the same-sex context, we observed sniffing behaviour only (the nose of one mouse directly contacted the body of the other mouse). In the opposite-sex contexts, we observed sniffing and mounting behaviour (the male placed his forelegs on the back of the female mouse). We, therefore, divided mouse vocalizations into three (MM and FF contexts) or four groups (MF contexts) according to the time of contact and the specific behaviour observed (figure 1*b*): (i) sniffing during the early phase (early: vocalizations during the early phase, sniffing only); (ii) sniffing during the middle phase (middle: vocalizations during the middle phase, sniffing only); (iii) sniffing during the late phase (late: vocalizations during the late phase, sniffing only); and (iv) mounting during the middle phase (mount: vocalizations during the middle phase, sniffing and three or more instances of mounting behaviour). We previously reported that male mice produced a similar type of calling bout during mounting in the middle and late phases of a context [18]. In this study, we also found a similar type of calling bout between these phases, but few animals displayed mounting behaviour in the late phase. Thus, we examined only vocalizations recorded during mounting in the middle phase. We compared the proportion of each call in the MM ((i) MM-early, (ii) MM-middle and (iii) MM-late), MF ((i) MF-early, (ii) MF-middle, (iii) MF-late and (iv) MF-mount) and FF ((i) FF-early, (ii) FF-middle and (iii) FF-late) contexts.

### 2.5. Statistical analysis

We used GraphPad Prism software (v. 7, San Diego, CA, USA) to perform one- and two-way ANOVAs followed by Tukey's test, tests for linear trends and Spearman correlation calculations. We used the mean values of call duration, RMS, peak frequency, fundamental frequency and entropy in each animal for statistical analysis, and compared these acoustic features among contexts or phases. We used multidimensional scaling (MDS) analysis to estimate similarities in vocalizations among all phases, according to the proportion of each call in all phases of each context. In addition, we used MDS analysis to estimate similarities in vocalizations between individuals, based on the proportion of each call throughout the entire context for all animals. These data are presented as MDS plots and dendrograms. The Euclidean distance was calculated using R software (v. 3.0.2, R Foundation for Statistical Computing; https://journal.r-project.org/; for more details, see the electronic supplementary material). We used a Spearman correlation test to assess whether the dimensions of the MDS plots were correlated with the proportions of several calls and several acoustic features. We used generalized linear mixed models to test the effect of observation time on the duration of recorded calls using R. We fitted Gamma-error distribution and log-link function for these models (for more details, see the electronic supplementary material). All data are presented as mean ± s.e.m.

## 3. Results

### 3.1. Vocalizations in same- and opposite-sex contexts

We analysed mouse ultrasonic vocalizations recorded during direct interaction tests in the MM, MF and FF contexts (total number of calls in MM: 5691 (*n* = 11), MF: 11 160 (*n* = 11) and FF: 8369 (*n* = 10)). We recorded vocalizations in the MM and MF contexts in 12 males, and those in the FF context in 12 females. However, we excluded data in one male and two females, because they did not produce vocalizations in all recording tests.

**Figure 2.**
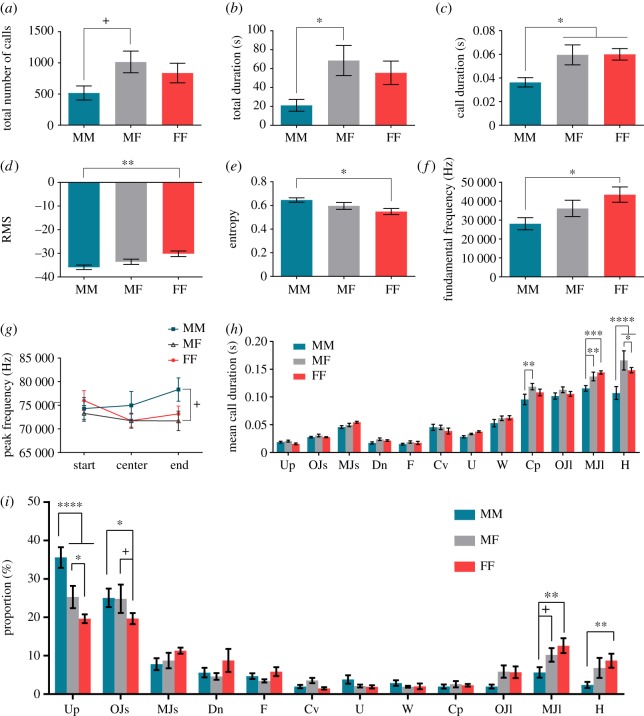
Differences in acoustic characteristics of mouse vocalizations among the MM, MF and FF contexts. (*a*) Total number of calls, (*b*) total duration, (*c*) call duration, (*d*) root-mean-square amplitude (RMS), (*e*) entropy, (*f*) fundamental frequency, (*g*) peak frequency of three points (start, centre and end) and (*h*) mean call duration of each call type. (*i*) Proportion of each call type in the MM, MF and FF contexts. Differences are denoted by an asterisk: ^+^*p* < 0.10, *p < 0.05, **p < 0.01, ***p < 0.001, ^#^*p* < 0.0001.

First, animals in the MF context emitted a greater number of calls (1015 ± 174.5 calls) and produced vocalizations for a longer period of time (68.58 ± 15.87 s) compared with those in the MM context (517.4 ± 112.5 calls, 21.13 ± 6.22 s) (figure 2*a*,*b*, *p *< 0.05). There are no significant differences between those in the MF and FF contexts (836.9 ± 158 calls, 55.57 ± 12.45 s).

Second, call durations in the MF (0.060 ± 0.0084 s) and FF (0.060 ± 0.0048 s) contexts were longer than those in the MM (0.036 ± 0.0039 s) context (figure 2*c*, *p *< 0.05). Particularly, the durations of the long calls (Complex, Multiple jumps long and Lower harmonics) in the MF and FF contexts were longer than those in the MM context (figure 2*h*, *p *< 0.05). Lower harmonics in the MF context were longer in duration than those in the FF context (figure 2*h*, *p *< 0.05).

Third, female vocalizations in the FF context showed larger RMS (−30.17 ± 1.18), lower entropy (0.55 ± 0.026) and a higher fundamental frequency (43 472 ± 4034 Hz) than male vocalizations in the MM context (RMS: −35.9 ± 0.95, entropy: 0.65 ± 0.018, fundamental frequency: 28 082 ± 3197 Hz) (figure 2*d*–*f*, *p* < 0.05). When comparing peak frequencies at the start, centre and end of each call among contexts, the peak frequency at the end in the MM context (78 334 ± 2459 Hz) was higher than that in the MF context (71 696 ± 2060 Hz) (figure 2*g*, *p* = 0.0648).

These results (call duration, RMS, entropy, fundamental frequency and peak frequency) might be related to the proportion of each call type. Specifically, animals in the MM context produced the highest rate of Upward calls (short calls with an upsweep in the frequency change, 35.59 ± 2.68%), followed by animals in the MF context (25.29 ± 2.88%) (figure 2*i*, *p* < 0.05). We suspect that this difference in the rate of Upward calls affected the peak frequency at the end of the calls. In addition, animals produced a greater number of One jump short calls in the MM and MF contexts (MM: 25.07 ± 2.39%, MF: 24.82 ± 3.69%) compared with those in the FF context (19.68 ± 1.43%) (figure 2*i*, *p* < 0.05). The usage of Upward and One jump short calls has been found to be similar in the MF context [18]. In the present study, we observed similar tendencies in the use of these calls among the contexts, suggesting that they have similar roles for communication and that they have overlapping roles among contexts. Conversely, the proportions of Multiple jumps long (12.61 ± 1.94%) and Lower harmonics (8.71 ± 1.82%) calls were higher in the FF compared with the MM context (Multiple jumps long: 5.65 ± 1.37%; Lower harmonics: 2.41 ± 0.79%) (figure 2*i*, *p* < 0.05). These long calls showed larger RMS values compared with short calls, and the RMS in the FF context were larger than those in the MM context (for more details, see the electronic supplementary material). These results indicate that, in same-sex contexts, male vocalizations contain a high rate of short calls, whereas female vocalizations contain a high rate of long calls. The proportions of long and short calls in the MF context occupied an intermediate position between those in the MM and FF contexts (figure 2*i*).

### 3.2. Within-subject vocalizations

**Figure 3.**
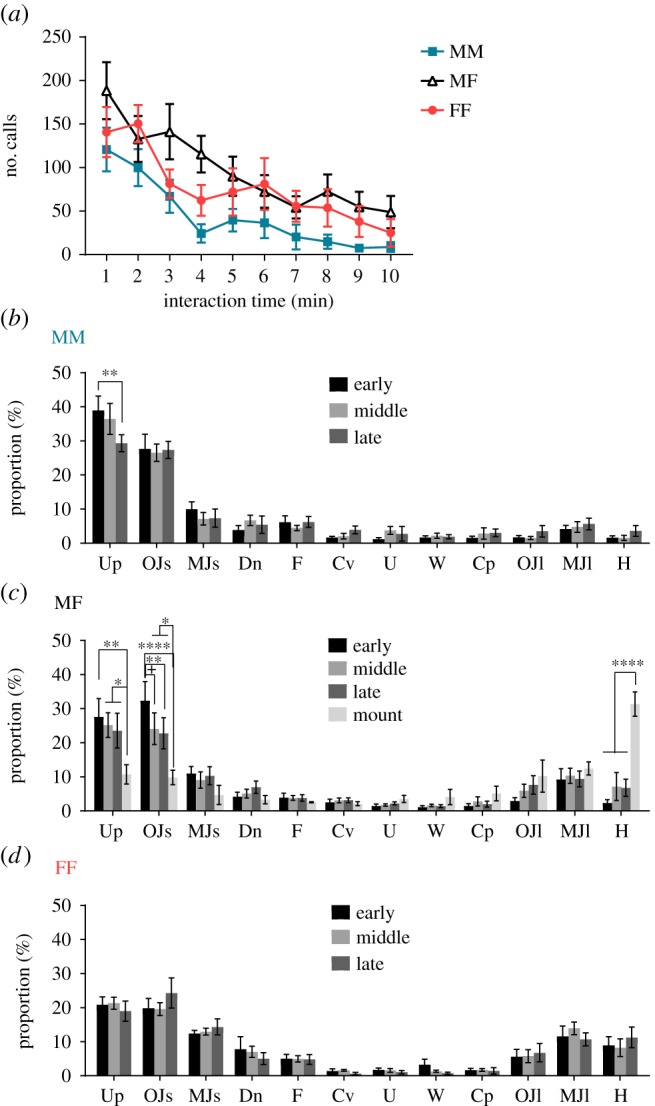
Number of calls produced per minute and proportion of each call type. (*a*) Number of calls per minute in the MM, MF and FF contexts. (*b*) The proportion of each call type in the early, middle and late phases of the MM context, (*c*) in the early, middle, late and mount phases of the MF context and (*d*) in the early, middle and late phases of the FF context. Differences are denoted by an asterisk: ^+^*p* < 0.10, *p < 0.05, **p < 0.01, ^#^*p* < 0.0001.

We previously found that vocalizations in the MF context changed depending on the contact duration and behaviour [18]. Likewise, in the present study, we grouped vocalizations according to interaction time and behaviour into the following phases: MM (MM-early: *n* = 10, MM-middle: *n* = 10, MM-late: *n* = 5), MF (MF-early: *n* = 9, MF-middle: *n* = 9, MF-late: *n* = 8, MF-mount: *n* = 3) and FF (FF-early: *n* = 9, FF-middle: *n* = 9, FF-late: *n* = 6). We recorded all phases in the MM and MF contexts in 12 males and those in the FF context in 12 females. Trials in which mice emitted fewer than 20 calls or showed only one to two instances of mounting behaviour were excluded from statistical analysis. Animals showed decreases in the number of calls with increasing interaction time, and we used calls for 1 min in the early phase and call for 3 min in middle, late and mount phases for analysis (mean number of calls in MM-early: 120.82 ± 25.16, MM-middle: 131.09 ± 35.83, MM-late: 31.36 ± 12.36, MF-early: 188.36 ± 32.71, MF-middle: 346.82 ± 69.36, MF-late: 176.45 ± 46.12, MF-mount: 385.33 ± 137.16, FF-early: 140.80 ± 28.82, FF-middle: 216.00 ± 46.49, FF-late: 116.80 ± 47.60; figure 3*a*).

The results in the MF context were similar to those in our previous study [18], although fewer mice displayed mounting behaviour. In the MF context, mice showed a high proportion of short calls (Upward: 27.60 ± 5.37%, One jump short: 32.36 ± 5.58%) in the early phase, long calls (MF-middle: Multiple jumps long: 10.34 ± 2.22%, Lower harmonics: 7.15 ± 4.11%; MF-late: Multiple jumps long 9.38 ± 2.32%, Lower harmonics: 6.79 ± 2.51%) in the latter phase, and Lower harmonics (Lower harmonics: 31.36 ± 3.56%) in the mount phase (figure 3*c*). This supports the hypothesis from our previous study that mice use different types of calling bouts according to the courtship phase.

By contrast, there are no significant time-based changes in most of the call proportions in the MM and FF contexts, although the proportion of Upward calls significantly decreased with contact time in the MM context (figure 3*b*,*d*). Males produced a higher proportion of short calls (MM-early: Upward: 38.94 ± 4.19%, One jump short: 27.64 ± 4.35%; MM-middle: Upward: 36.44 ± 4.56%, One jump short: 26.57 ± 2.53%; MM-late: Upward: 29.32 ± 2.48%, One jump short: 27.35 ± 2.50%) throughout the MM context (figure 3*b*), while females produced long calls (FF-early: Multiple jumps long: 11.57 ± 3.03%, Lower harmonics: 8.95 ± 2.51%; FF-middle: Multiple jumps long: 13.93 ± 1.87%, Lower harmonics: 8.24 ± 2.60%; FF-late: Multiple jumps long: 10.71 ± 1.92%, Lower harmonics: 11.26 ± 3.01%) throughout the FF context (figure 3*d*).

**Figure 4.**
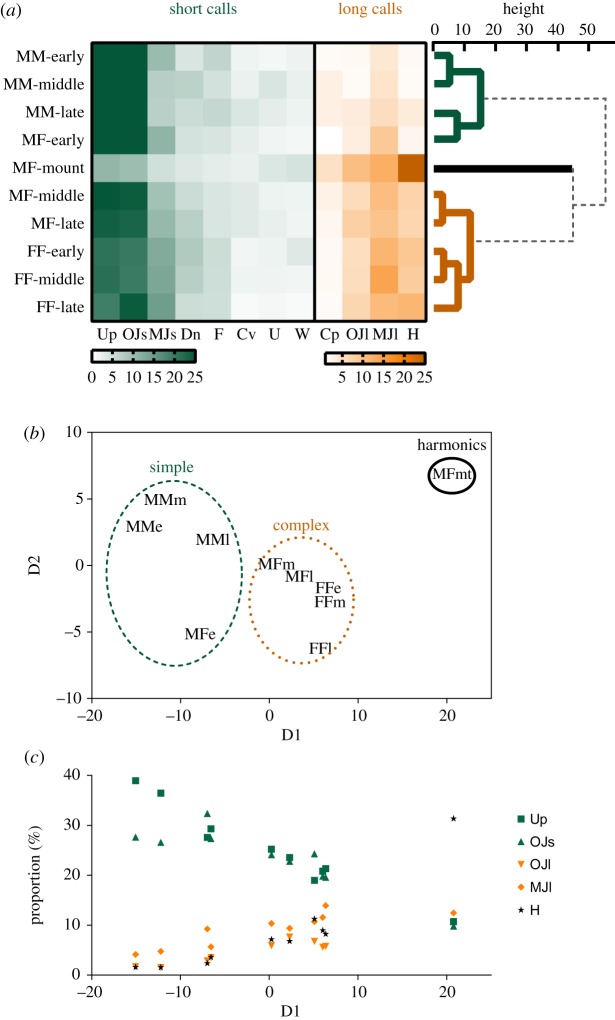
Cluster dendrogram and MDS plot showing the structure of vocalizations produced in each phase in all contexts. (*a*) Cluster dendrogram with heatmap showing the proportion of each call type in all phases (MM-early, MMe; MM-middle, MMm; MM-late, MMl; MF-early, MFe; MF-middle, MFm; MF-late, MFl; MF-mount, MFmt; FF-early, FFe; FF-middle, FFm; FF-late, FFl). (*b*) MDS plot showing the similarities in the proportion of each call type among all the phases and (*c*) the proportion of Up, OJs, OJl, MJl and H calls in each phase in all contexts.

Figure 4 shows a cluster dendrogram with a heatmap, and MDS plots showing the proportion of each phase in all contexts (figure 4*a*,*b*). The calling bouts could mainly be divided into three groups: (i) calling bouts composed predominantly of short calls (Simple), (ii) calling bouts with a high proportion of long calls (Complex) and (iii) calling bouts composed predominantly of Lower harmonics (Harmonics) (figure 4*b*,*c*). Dimension 1 (D1) on the MDS plot was highly correlated with the Upward (*r* = −0.98, *p* < 0.0001), One jump short (*r* = −0.88, *p* < 0.001), One jump long (*r* = 0.95, *p* < 0.0001), Multiple jumps long (*r* = 0.86, *p* < 0.01) and Lower harmonics (*r* = 0.90, *p* < 0.001) calls (figure 4*c*). In all phases of the MM context and the early phase of the MF context (MM-early, MM-middle, MM-late and MF-early), animals produced Simple calling bouts. Conversely, in all phases of the FF context and the middle and late phases of the MF context (FF-early, FF-middle, FF-late, MF-middle and MF-late), animals produced Complex calling bouts. During mounting behaviour in the MF context (MF-mount), animals produced Harmonics calling bouts, which differ substantially from other calling bouts. Therefore, males produced Simple calling bouts and females produced Complex calling bouts in the same-sex context, while mice produced Simple, Complex and Harmonics calling bouts in the opposite-sex context. This result suggests that mice can use different types of calling bouts according to the context and phase of the context, and that several types overlap among same-sex and opposite-sex contexts.

### 3.3. Between-subject vocalizations

**Figure 5.**
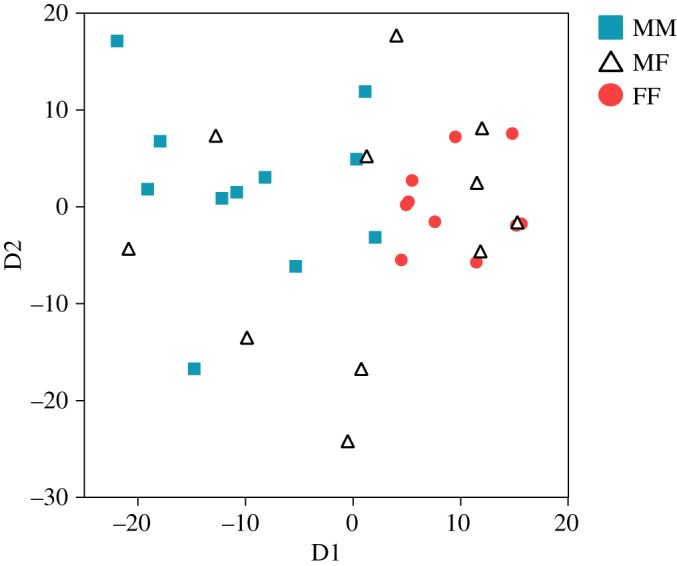
MDS plot showing the similarities in the proportion of each call type among individuals. The D1 was highly correlated with the quantity and quality of calls.

Figure 5 contains an MDS plot that shows the degree of similarity of the proportion of each call type in each mouse (MM: *n* = 11, MF: *n* = 11 and FF: *n* = 10; figure 5). D1 was highly correlated with the rate of Upward (*r* = −0.90, *p* < 0.0001), One Jump short (*r* = −0.47, *p* < 0.01), One jump long (*r* = 0.38, *p* < 0.05), Multiple jumps short (*r* = 0.46, *p* < 0.01), Multiple jumps long (*r* = 0.78, *p* < 0.0001) and Lower harmonics (*r* = 0.65, *p* < 0.0001) calls. In addition, D1 was highly correlated with the total number of calls (*r* = 0.59, *p* < 0.001), call duration (*r* = 0.71, *p* < 0.0001) and RMS (*r* = 0.62, *p *< 0.001). These findings reflect the amount and complexity of the vocalizations. Most vocalizations from the MM context are on the left side of the MDS plot, while most vocalizations from the FF context are on the right side. This indicates that most males produced Simple calling bouts and most females produced Complex calling bouts in the same-sex context. Meanwhile, in the opposite-sex context, the complexity of vocalizations varied between individuals.

## 4. Discussion

In this study, we recorded and compared mouse vocalizations in same- and opposite-sex contexts. First, we found that male mice typically produced Simple calling bouts in the male–male context, and female mice typically produced Complex calling bouts in the female–female context. Mice produced both Simple and Complex calling bouts in the male–female context. Second, we observed larger between-subjects variability in vocal complexity in the male–female context compared with that in same-sex contexts. This indicates that the usage of calling bout varies according to sociosexual context. Specifically, the observed variability in the male–female context was likely caused by differences in the proportions of the three types of calling bouts.

### 4.1. Calling bouts overlapped among contexts

We found that male and female mice produced different types of calling bouts in the same-sex context. Specifically, male mice produced Simple calling bouts and female mice produced Complex calling bouts. This dissimilarity is likely linked to sex differences in behaviour in a natural environment. Males, especially dominant males, form a territory and show territorial behaviour, including aggressive behaviour, towards intruder animals [23–26]. Vocalizations in animals with territories may take the form of Simple calling bouts. Simple calling bouts mainly consist of short calls (87.17 ± 1.77%). Chabout *et al*. [11] reported that isolated males, but not mice maintained in a group, produced most short calls during social contact with novel male. This suggests that isolated male mice form a territory and communicate using short calls towards intruder as one of territorial behaviour. By contrast, females form social groups, and interact and cooperate with others in a communal nest [27–30]. Complex calling bouts may be useful in maintaining the group structure. Specifically, studies examining female vocalizations using a resident–intruder paradigm have suggested that female vocalizations are important for establishing dominance in the community hierarchy [31]. Female mice produce more vocalizations towards novel versus familiar females [22,31–33]. In addition, feeding state, the oestrus cycle, pregnancy and age also affect vocalizations [15,22]. Female vocalizations in the female–female context may communicate information about state and may be important for cooperative behaviour such as foraging and feeding.

We also found that Simple and Complex calling bouts were produced in the male–female context. Mice produced Simple calling bouts in the early phase, at which point the mouse first contacted the novel animal. As in the male–male context, this type of calling bout may be used by animals with territories. The vocalizations generated when a male mouse interacts with a female mouse have been found to include a higher proportion of short calls, which Simple calling bouts mainly consist of, compared with those observed when a male mouse sniffs female urine [10]. This suggests that mice produce short calls when interacting with novel animals. In addition, the proportion of short calls produced in the male–female context increased after a female was removed from the test cage [34]. Male mice are known to produce short calls during exploratory behaviour in non-social contexts, and they exhibit increased production of short calls in novel environments [11,35]. Therefore, male mice may produce short calls when they interact with novel intruder animals and novel environments, and not just other male mice. Animals in both male–male and male–female contexts, especially in the presence of isolated males, may produce Simple calling bouts towards intruder males and novel animals as one of territorial behaviour.

Conversely, animals produced Complex calling bouts in the middle and late phases. Animals in these phases in the male–female context produced a greater number of vocalizations compared with those in the male–male context, and the male eventually displayed approach behaviour towards the female during courtship. Complex calling bouts contained various calls and had a high proportion of long calls (27.91 ± 0.88%) compared with Simple calling bouts (12.83 ± 1.77%). A previous study found that, in the male–female context, animals produced a higher proportion of long calls when the female was awake versus anaesthetized [2], indicating that these long calls play a social role. In our previous study, amygdala-lesioned mice showed decreased physical contact, mounting behaviour and production of long calls in a male–female context [36]. Another study found that female mice prefer vocalizations featuring long versus short calls [10]. In this study, mice appeared to produce Complex calling bouts with approach behaviour in both the female–female and male–female contexts. Thus, this type of calling bout may facilitate the establishment of relationships in same-sex (female only) and opposite-sex contexts.

Simple and Complex calling bouts took the form of continuous vocalizations in the male–female context and could not be divided clearly with behavioural changes in the present study. However, it is possible that each type of calling bout reflects differences in internal state, and has a phase-specific role. These possibilities should be examined in future research.

Although we were not able to distinguish which of the two mice produced ultrasonic sounds, identifying the individual emitters will contribute to the understanding of ultrasonic communication, especially in the male–female context. Previously, vocalizations in male–female contexts were thought to be produced by male mice only, but recent studies have shown that females also produce vocalizations. For example, females have been shown to produce ultrasonic vocalizations with a comparable quality and quantity as males when encountering males, but are separated such that they are unable to make physical contact [16]. Another study using a microphone array in which to locate the sound source found that females produced vocalizations when males exhibit courtship behaviour in a socially interacting group [37]. Therefore, it is likely that in our experiments, both males and females produced ultrasonic sounds in the opposite-sex context. In the same-sex context, males produced Simple calling bout, whereas females produced Complex calling bout. These two types of calling bouts were also recorded in male–female contexts, suggesting that, like in the same-sex contexts, males and females produced Simple and Complex bouts, respectively. However, it is possible that the male produced not only Simple but also Complex calling bouts in male–female context. This possibility stems from previous studies showing that overall vocal production does not change dramatically whether a male–female interaction involves a vivid or anaesthetized female [2]. In addition, a study using a microphone array shows that males in a socially interacting group produce the majority of vocalizations during courtship [37]. Male mice deficient for TRP2, which is known to be related to the sensory activation in the vomeronasal organ, produce a similar amount of mounting and ultrasonic vocalizations towards males and females [38]. Our results suggest that mice have the capacity to produce Simple and Complex calling bouts, and they produce different types of calling bouts according to phase and context. In future studies, we hope to detect which mice produce the vocalizations, to further examine the function of calling bouts in sociosexual contexts.

### 4.2. Harmonics calling bout is specific to the mounting phase

Lower harmonics are complex calls with a long duration, high RMS and harmonic tone of approximately 30–40 kHz. This call type is reportedly related to mounting behaviour [3,18,19,36]. We previously reported that the number of Lower harmonics was highly correlated with the number of mounting instances and that amygdala lesions in males affected a number of mounting behaviours and the vocalization of longer calls, especially Lower harmonics calls (unpublished data) [36]. In the present study, mice in the male–female and female–female contexts produced calls with similar durations for most of the call types, with the exception of Lower harmonics. The mean duration of the Lower harmonics call in the male–female context was longer than that in the female–female context. In addition, the Harmonics calling bout consisting of repeated Lower harmonics calls was not observed in the same-sex contexts. These results suggest that this type of calling bout was specific to the male–female context, especially the phase featuring mounting. This type was reportedly produced during mounting, but not during intromission, suggesting that this type is associated with female responses [18]. Future examinations of vocalizations associated with female responses will contribute to our understanding of ultrasonic communication in mouse courtship behaviour.

### 4.3. Individual differences in vocal complexity

We considered the quantity and quality of vocalizations in the male–female context to be deeply associated with the sexual experiences and sexual motivation of the males towards the female mice.

We found no significant differences in the number of vocalizations between the male–female and female–female contexts. These results were inconsistent with those of a previous study, which reported that mice produced a significantly greater number of vocalizations in the female–female context compared with the male–female context [2]. This discrepancy might be due to differences in the sexual experience of the male mice. The animals in our study had sexual experience prior to the recording test, while the male mice in the previous study did not. Vocalizations in the male–female context are reportedly affected by various factors, such as mouse strain, growth environment, the oestrus cycle of the female and the sexual experience of the male [3,11,39–42]. However, in the present study, experimental animals were controlled in terms of genetic variation, and we controlled the housing conditions and the oestrus cycle of the exposure females. Our results support the hypothesis that social experience with a member of the opposite sex is related to male sexual motivation towards females and that it dramatically influences motivation to emit ultrasonic vocalizations in animals [41].

This sexual motivation could affect not only the number of vocalizations, but also the vocal complexity in the male–female context. This has been suggested in several previous studies. Androgen is known to be related to male sexual behaviour and to the number of vocalizations [43]. Testosterone, which is a major androgen, reportedly affected the proportion of calls with simple sweeps in monogamous California mice [44]. An audience effect has recently been reported in mice, wherein mice produce more complex calls in the presence of additional listeners [45]. In the present study, we found that the amount and complexity of vocalizations in the male–female context varied widely between individuals, while this was not the case in the same-sex context. In addition, the number of vocalizations, which has been found to be affected by sexual experience and androgen [40,41,43], was highly correlated with the proportion of long calls in the present study (*r* = 0.743, *p* < 0.01). These results suggest that the large variation in vocal complexity may reflect differences in male sexual motivation towards female mice. Males with high sexual motivation might approach females initiatively and carry the phases further with complex sounds, while males with low sexual motivation might show territorial behaviour and spend more time in the first phase with simple sounds. If this assumption holds true, the observed individual differences in vocalizations in the male–female context may be an index of male sexual motivation regarding the female intruder mice. In future work, we plan to examine the relationship between the state of the animal and the proportion of each type of calling bout, and to assess the role of each type in vocal communication by examining listeners' responses to specific vocalizations.

### 4.4. Comparison between sociosexual contexts

**Figure 6.**
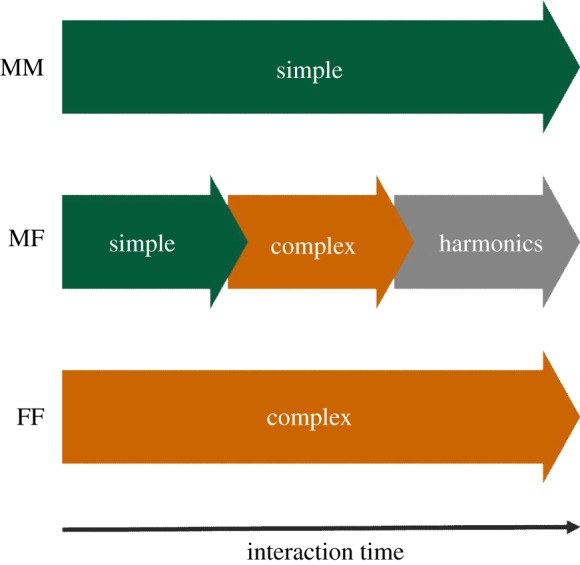
Summarized diagram showing the usage of calling bouts in the MM, MF and FF contexts.

In summary, mice can produce three types of calling bouts: Simple, Complex and Harmonics (figure 6*a*). These vocalizations overlapped among opposite-sex and same-sex contexts (figure 6*a*), suggesting that the usage of these calling bouts was affected by motivation with respect to intruders, and that the roles of these types of calling bouts overlapped among the contexts. Our findings support the view that vocalizations in the male–female context function not only as part of courtship, but that they also play a part in other types of communication, including territorial behaviour [2,16]. These findings contribute to our understanding of ultrasonic communication in mice and represent a platform for future studies using genetically modified mice to examine social behaviour in human psychiatric disorders.

**Figure 7.**
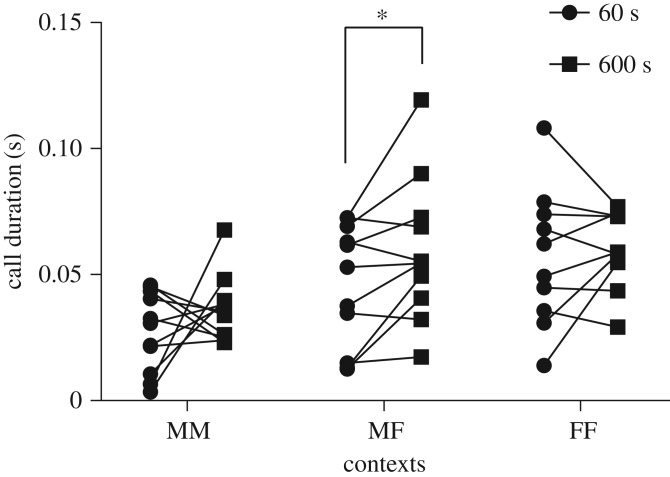
Call duration in the first 60 and 600 s of the MM, MF and FF contexts. Differences are denoted by an asterisk: *p < 0.05.

Moreover, our study demonstrates the importance of recording duration for analysis. Many studies regarding mouse vocalizations have been published recently, and these vary widely in terms of methods, especially recording duration. Recording mouse vocalizations with inappropriate durations may have a number of consequences. For example, figure 7 shows the mean call duration, whose acoustic feature is often used to compare the vocalizations in mice, in the first 60 and 600 s of each context. Call duration in each same-sex context was not different between first 60 and 600 s (MM: *χ*^2^ = 1.41, d.f. = 1, *p = *0.23; FF: *χ*^2^ = 0.99, d.f. = 1, *p = *0.32), whereas it was different in the male–female context (*χ*^2^ = 6.43, d.f. = 1, *p < *0.05). These findings can be explained by examining the phases and phase-specific types of calling bouts comprising vocalizations that differ depending on the duration of interaction.

We also considered individual differences in vocalization in the different sociosexual contexts. We found that while most mice could produce the same call types, the amount and complexity of the vocalizations varied widely in the male–female context. The main structure of mouse vocalizations depends on the strain, and previous studies have revealed that the main structure is genetically determined. However, several acoustic structures are influenced by the environment. In a previous study comparing vocalizations in wild and laboratory California mice, the mice in both groups produced vocalizations with a similar structure, but the frequency variance in the wild mice was higher than that in the laboratory mice [46]. As mentioned, the number and frequency of mouse vocalizations are affected by social experiences, including sexual experience [11,39,41]. These results suggest that the environment, especially social experiences, can modulate the quantity and quality of vocalizations. This variability may explain inconsistencies in the results of studies that compared different sociosexual contexts. The experimental design must be carefully considered when directly comparing vocalizations between several conditions.

## 5. Conclusion

In this study, we recorded ultrasonic vocalizations during same- and opposite-sex contexts in male and female mice. Mice produced three types of calling bouts, and two of these calling bouts overlapped in different social contexts. This suggests that some context phases with phase-specific types of calling bouts overlapped among the contexts, such that each type of calling bout has a phase-specific role. Additionally, we found that the complexity of the vocalizations in the male–female context varied widely between individuals. The complexity of vocalizations may communicate the state and reflect level of sexual motivation. Our results contribute to the characterization of mouse ultrasonic vocalizations and understanding of mouse ultrasonic communication. Furthermore, our findings may be useful in developing a criterion for evaluating social behaviour.

## Supplementary Material

Call parameter

## Supplementary Material

Data

## Supplementary Material

R data

## Supplementary Material

R code

## Data accessibility

The datasets supporting this article, call parameter, and code and data for analyses in R have been uploaded as part of the electronic supplementary material.
